# The complete chloroplast genome sequence of *Rosa minutifolia*

**DOI:** 10.1080/23802359.2020.1817807

**Published:** 2020-09-08

**Authors:** Xia Zhao, Chengwen Gao

**Affiliations:** a Department of Hematology, The Affiliated Hospital of Qingdao University, Qingdao, China; b Laboratory of Medical Biology, The Affiliated Hospital of Qingdao University, Qingdao, China

**Keywords:** Chloroplast genome, *Rosa minutifolia*, Illumina sequencing, phylogenetic analysis

## Abstract

Here we report the chloroplast genome sequence of *Rosa minutifolia*. This rose grows in warm climates, it has been reported as an endangered species. The complete chloroplast genome size was 157,396 bp, and exhibited a quadripartite structure with dual inverted repeats (IRs) regions (26,041 bp), a small single-copy (SSC) region (18,775 bp), and a large single-copy (LSC) region (86,539 bp). The genome contained a total of 137 genes including 90 protein-coding genes, 39 tRNA genes, and 8 rRNA genes. Phylogenetic analysis revealed that *R. minutifolia* is closely related to *R. rugosa* and *R*. hybrid cultivar Augusta.

*Rosa minutifolia* is a species of rose known by the common names Baja rose, Desert Rose and small-leafed rose. This is a very spiny, dense shrub native to the chaparral plant community of Baja California and San Diego County, California (Lewis 2016; Bruneau et al. 2007).

This rose grows in warm climates. It tends to be quite dwarf in dry habitats. Produces bright pink single flowers. Rose is very thorny with thorns very close together and intense. Natural habitat is coastal sage scrub with a desert feeling. Very good rose for the rock garden and cactus garden. This rose reported to be an endangered species in the U.S. but plants still present in Mexico. In this study, we report the chloroplast genome sequence of *R. minutifolia* to provide the underlying information for genetic breeding and conservation studies of this species.

Total DNA (Voucher specimen: N48.85°, E2.35°, INRA, SAMN08979059) was isolated using a modified CTAB method (Allen et al. 2006) and sequenced by the Illumina HiSeq 2500 (Illumina Inc., San Diego, CA) platform with pair-end library. Trimmomatic v0.38 (Bolger et al. 2014) was used to filter the raw sequencing data. The resultant clean data was used to perform de novo assembly using SPAdes 3.61(Bankevich et al. 2012) with varying K-mer parameters. We ordered *de novo* scaffolds that were positively correlated to the chloroplasts on to the reference chloroplast genome of *R. roxburghii* (NC_032038). Next, the Geneious Prime software v2020.0.4 (Kearse et al. 2012) was used to remap the reads to fill gaps in the final consensus sequence with multiple iterations. GeSeq (Tillich et al. 2017) was used to conduct chloroplast genome annotation to predict gene-encoding proteins, transfer RNA (tRNA), and ribosomal RNA (rRNA) with manual adjustments as required. We manually examined the IR junctions of *R. minutifolia*. The complete chloroplast genome sequence together with gene annotations were submitted to the GenBank with the accession number of MT755634.

The complete chloroplast genome of *R. minutifolia* has a total length of 1,57,396 bp, and exhibited a quadripartite structure with dual IR regions (26,041 bp), an SSC region (18,775 bp), and an LSC region (86,539 bp). *R. minutifolia* contained 137 genes in its plastid genome, of which, 90 were protein-coding genes, 8 were rRNAs, and 39 were tRNAs. Sixteen genes exhibited introns, of which, six tRNAs (*trnV-UAC, trnG-GCC, trnK-UUU, trnL-UAA, trnA-UGC* and *trnI-GAU*) and ten protein-coding genes (*rps16, rpl16, ycf3*, *rps12*, *ndhB, petB, rpl2, rpoC1, ndhA* and *clpP*). Furthermore, The GC content of *R. minutifolia* chloroplast genome was 37.21%.

To further investigate its taxonomic status, a Maximum-likelihood (ML) tree was constructed based on complete chloroplast genome sequences using MEGA 7.0 (Kumar et al. 2016) with 1000 bootstrap replicates. Here, the sequences of *Rosa* chloroplast genomes were aligned using MAFFT 7.221 (Katoh and Standley 2013). Chloroplast genome sequences of 15 species in *Rosa* (*R. minutifolia, R. lucieae, R. multiflora, R. banksiae,R. chinensis* var. *spontanea,R. hybrid cultivar, R. berberifolia, R. canina, R. laevigata, R. laevigata* var. *leiocarpa, R. praelucens, R. rugosa, R. maximowicziana, R. roxburghii, R. xanthina*) and *Geum rupestre* as the outgroup. The phylogenetic analysis revealed that *R. minutifolia* is closely related to *R. rugosa* and *R*. hybrid cultivar Augusta (Figure 1). This newly determined chloroplast genome sequence will provide new genetic resource for studies of *R. minutifolia*.

**Figure 1.**
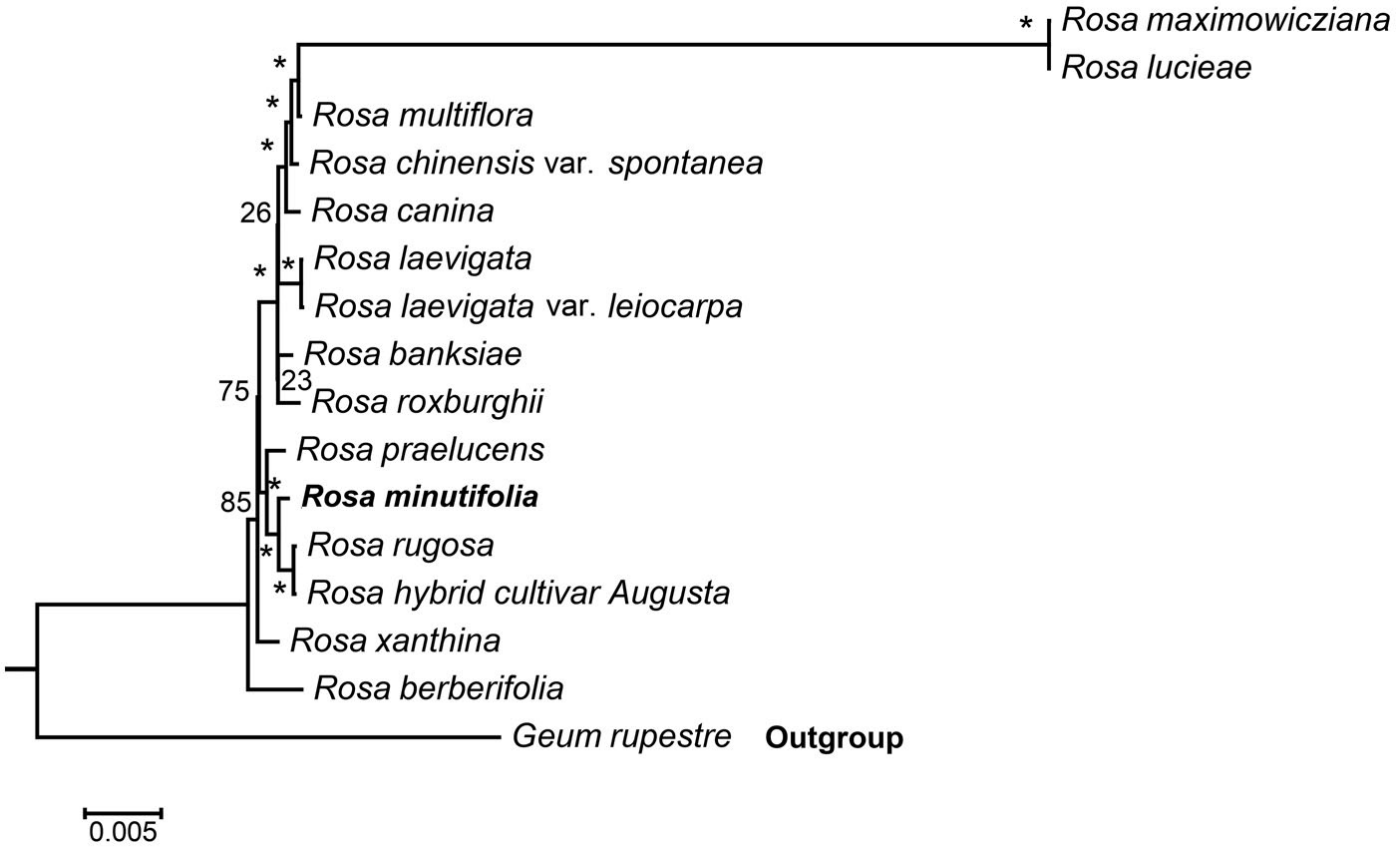
Maximum likelihood phylogenetic tree of *R. minutifolia* with 15 species in the order *Rosa* based on complete chloroplast genome sequences. Numbers in the nodes are bootstrap values from 1000 replicates. The chloroplast sequence of *Geum rupestre* was set as the outgroup. Accession numbers are as below: *R. minutifolia* (MT755634), *R. banksiae* (NC_042194)*, R. berberifolia* (NC_045126), *R. canina* (NC_047295),*R. hybrid cultivar* (NC_044126), *R. laevigata* (NC_046824), *R. laevigata* var. *leiocarpa* (NC_047418), *R. chinensis* var. *spontanea* (NC_038102),*R. lucieae* (NC_040997), *R. multiflora* (NC_039989)*, R. praelucens* (NC_037492)*, R. rugosa* (NC_044094), *R. maximowicziana* (NC_040960), *R. roxburghii* (NC_032038)*, R. xanthina* (MT547539), *Geum rupestre* (NC_037392).

## Data Availability

The data that support the findings of this study are openly available in GenBank of NCBI at https://www.ncbi.nlm.nih.gov, reference number MT755634. Chloroplast genome raw data have been deposited at the NCBI Sequence Read Archive (SRA) under accession PRJNA657453.
